# Calcium-Dependent Protein Kinase in Ginger Binds with Importin-α through Its Junction Domain for Nuclear Localization, and Further Interacts with NAC Transcription Factor

**DOI:** 10.3389/fpls.2016.01909

**Published:** 2017-01-13

**Authors:** Padmanabhan Jayanthi Vivek, Mohankumar Saraladevi Resmi, Sweda Sreekumar, K. C. Sivakumar, Narendra Tuteja, Eppurathu Vasudevan Soniya

**Affiliations:** ^1^Rajiv Gandhi Centre for BiotechnologyThiruvananthapuram, India; ^2^Amity Institute of Microbial Technology, Amity UniversityNoida, India

**Keywords:** calcium-dependent protein kinase, ginger, importin, nuclear localization sequence, transcription factor

## Abstract

Calcium-dependent protein kinases (CDPKs) are important sensors of Ca^2+^ elevations in plant cells regulating the gene expression linked with various cellular processes like stress response, growth and development, metabolism, and cytoskeleton dynamics. Ginger is an extensively used spice due to its unique flavor and immense medicinal value. The two major threats that interfere with the large scale production of ginger are the salinity and drought stress. ZoCDPK1 (*Zingiber officinale* Calcium-dependent protein kinase 1) is a salinity and drought-inducible CDPK gene isolated from ginger and undergoes dynamic subcellular localization during stress conditions. ZoCDPK1, with signature features of a typical Ca^2+^ regulated kinase, also possesses a bipartite nuclear localization sequence (NLS) in its junction domain (JD). A striking feature in ZoCDPK1 is the rare occurrence of a coupling between the NLS in JD and consensus sequences in regulatory domain. Here, we further identified its nature of nuclear localization and its interaction partners. In the homology model generated for ZoCDPK1, the regulatory domain mimics the crystal structure of the regulatory domain in *Arabidopsis* CDPK1. Molecular docking simulation of importin (ZoIMPα), an important protein involved in nuclear translocation, into the NLS of ZoCDPK1 was well-visualized. Furthermore, the direct interaction of ZoCDPK1 and ZoIMPα proteins was studied by the yeast 2-hybrid (Y2H) system, which confirmed that junction domain (JD) is an important interaction module required for ZoCDPK1 and ZoIMPα binding. The probable interacting partners of ZoCDPK1 were also identified using Y2H experiment. Of the 10 different stress-related interacting partners identified for ZoCDPK1, NAC transcription factor (TF) needs special mention, especially in the context of ZoCDPK1 function. The interaction between ZoCDPK1 and NAC TF, in fact, corroborate with the results of gene expression and over-expression studies of ZoCDPK1. Hence ZoCDPK1 is operating through NAC TF mediated ABA-independent, cold non-responsive stress signaling pathway in ginger.

## Introduction

Salinity, drought, extreme temperatures, chemical toxicity, pathogen infection, and oxidative stress are serious threats to agriculture and the natural status of the environment (Easterling et al., 2007; Fischer and Edmeades, 2010; Vivek et al., 2013). Responses to different stress stimuli could be achieved through an increase in intracellular Ca^2+^ concentrations and relay of divergent Ca^2+^ signal transduction cascades (Kudla et al., 2010). Specific Ca^2+^ signatures may be recognized by different sensor proteins. Three major families of Ca^2+^ sensors have been identified in higher plants: Calmodulins (CaMs) and CaM-like proteins (Perochon et al., 2011), calcineurin B-like (CBL) proteins (Luan, 2009), and calcium-dependent protein kinases (CDPKs) (Deinlein et al., 2014). Among them, CDPKs are the best characterized and are unique in plants. CDPKs are composed of a Ca^2+^ sensing and a protein kinase effector domain in a single protein and thus represent a key player for both perception and signal propagation of intracellular Ca^2+^ changes upon various stimuli (Deinlein et al., 2014).

CDPKs are specific to the plant kingdom and some protozoans but absent in animals and fungi. They comprise a protein family with 34 members in *Arabidopsis thaliana* (Hrabak et al., 2003) and 31 members in *Oryza sativa* (Ray et al., 2007). All family members share a conserved modular structure, consisting of an N-terminal variable domain (NV), a highly conserved serine/threonine protein kinase effector domain (KD), a junction domain (JD), a regulatory or Ca^2+^-sensing calmodulin-like domain (CaM-LD) with four Ca^2+^-binding EF-hand motifs and a C-terminal variable (CV) domain (Harmon, 2003). The N-terminal domains of CDPKs vary in length from 40 to 180 amino acids, and there is no significant homology in the sequence even between family members (Harmon, 2003). The JD between the kinase and CaM-LD functions as a pseudo-substrate autoinhibitor that inhibits phosphorylation in the absence of Ca^2+^ and keeps the CDPK in a state of low activity (Harmon et al., 1994). The CaM-LD of CDPKs consist of two structural domains (termed the N and C “lobes”), each containing two EF hand helix-loop-helix Ca^2+^− binding motifs. Activation of CDPK occurs when Ca^2+^ levels rise to fill the two weaker affinity-binding sites in the N-lobe, thereby triggering a conformational change that leads to release of autoinhibitory region (Christodoulou et al., 2004). CDPKs are found in multiple locations including the cytosol, nucleus, plasma membrane, endoplasmic reticulum, peroxisomes, mitochondrial outer membrane, and oil bodies (Harper et al., 2004; Vivek et al., 2013). Dehydration, chilling temperature, salinity and hormones can all induce specific changes in the expression of CDPK genes in *Arabidopsis*, rice, tobacco, and wheat (Yoon et al., 1999; Ma and Wu, 2007; Wan et al., 2007; Li et al., 2008; Franz et al., 2011; Vivek et al., 2013).

Ginger is a widely utilized spice crop not only for enhancing the flavor and aroma of food but also for treating diverse diseases. Salinity and drought stress are serious impediments in the commercial cultivation of ginger (Vivek et al., 2013; Ajav and Ogunlade, 2014). We have reported earlier that ZoCDPK1, a stress-inducible CDPK in ginger, functions in the positive regulation of the signaling pathways that are involved in the response to salinity and drought stress. Its over-expression has shown to provide salinity and drought tolerance in tobacco (Vivek et al., 2013). An interesting feature of ZoCDPK1 is the presence of a bipartite NLS in its JD and hence it can interact with nuclear transporting proteins for its nuclear localization. This was further supported by the predominant nuclear localization of ZoCDPK1 (Vivek et al., 2013). The importin-α/β heterodimer targets hundreds of proteins to the nuclear-pore complex (NPC) and facilitates their translocation across the nuclear envelope. Importin-α binds to NLS-containing proteins and links them to importin-β, and translocate to the nucleus (Goldfarb et al., 2004). Importin-αs are composed of a flexible N-terminal importin-β-binding (IBB) domain and a highly structured domain comprised of ten tandem armadillo (ARM) repeats (Fontes et al., 2000). The helical ARM repeats assemble into a twisted slug-like structure, whose belly serves as the NLS-binding groove.

In order to understand the possible ginger CDPK1 regulated pathway, it is very much essential to identify the possible interaction partners of ZoCDPK1 gene. Here we report that the predominant nuclear localization of ZoCDPK1 is through the interaction between NLS containing domain of the CDPK (JD) and importin-α protein (ZoIMPα). We extended this finding with *in silico* docking studies of ginger CDPK1 with importin-α. To know further about the proteins involved in this signal transduction, we have studied the interaction of ZoCDPK1 with ginger cDNA library.

## Materials and methods

### Homology modeling

The sequence of ZoCDPK1 was subjected to a homology search using BLAST and PSI-BLAST against NCBI PDB database. No full length template was obtained for modeling the sequence. As it was found that the protein template 2BDW (kinase domain of *Caenorhabditis elegans* CaMKII) and 2AAO (regulatory domain of *A. thaliana* CDPK1) showed significant sequence identity with the kinase domain and CaM-LD of ZoCDPK1 respectively, their PDB co-ordinates were retrieved from Protein Data Bank (http://www.rcsb.org/pdb/). Homology modeling was carried out using MODELLER9 (Sali and Blundell, 1993) based on the sequence alignment generated between template and target sequences. The atomic coordinates were obtained from template structures for modeling ZoCDPK1. Energy minimization of the top scored model was carried out with GROMACS 3.3.1 (Van Der Spoel et al., 2005) using OPLSAA force field. The minimization was set to run for 5000 steps or until convergence to machine precision. PROSA2003 (Wiederstein and Sippl, 2007) programme was used for validation of the model, by analyzing residue interaction energy and *Z*-score. These procedures were iterated several times until a good quality model was obtained.

### *In silico* protein-protein interaction

A protein-protein interaction docking was performed for ZoCDPK1 with importin-α using ZDOCK and RDOCK (Wiehe et al., 2005) installed locally. The structure of importin-α was taken from PDB (1PJN), in which an NLS peptide complexed with the protein was removed before docking study. Since it was expected that the NLS region in the JD of ZoCDPK1 should interact with the NLS peptide binding residues found in importin, those residues were specified for filtering in ZDOCK. RDOCK refinement was performed on the top 10 poses of the filtered ZDOCK output. The top scored model obtained from RDOCK was used as a starting point for local searches using the RosettaDock server (Lyskov and Gray, 2008) in which 10000 structures were independently calculated and energetically scored. The energetically best structure complex was filtered and subjected to further investigations. Visualization of structures was accomplished with PyMol 0.99rc6 (http://www.pymol.org/). The electrostatic potential on the surface of the proteins was calculated by solving the Poisson–Boltzmann equation using the program APBS (Baker et al., 2001).

### Cloning of importin-α (ZoIMPα) from ginger

Nucleotide sequences corresponding to importin-α were retrieved from ginger EST database and aligned together to get the full length information. In order to get the full length gene IMPF (5′-GAGCGCCGCCATGGAGATGTCGCTCCGGCCGAGCGAG-3′) and IMPR (5′- TCGAGCTCGATGGATCCCGCTGAAGTCGAATCCACCA-3′) primers were designed from this sequence information and PCR was carried out using cDNA of ginger. The PCR was carried out in a 50 μL reaction mixture containing 1.0 μL of cDNA, 5x Phusion High-Fidelity Reaction Buffer, 200 μM of each dNTPs, 0.6 μL of Phusion DNA Polymerase (2 U/μL; NEB, England) and 20 pmol each of IMPF and IMPR. The reaction mixture was denatured at 98°C for 30 s followed by 35 cycles (98°C for 10 s, 60°C for 30 s, 72°C for 1 min) and a final incubation at 72°C for 10 min. The product was cloned into pGEM®-T Easy vector and sequenced using T7 and Sp6 primers. The nucleotide and amino acid sequences of ZoIMPα were subjected to analysis using BLASTN, PSI-BLAST, and PHI-BLAST.

### Yeast two-hybrid experiment

A Gal4-based yeast two-hybrid system was used to study (i) the interaction of ZoCDPK1 with ginger importin-α and (ii) the interaction of ZoCDPK1 with ginger Y2H library proteins. For ZoCDPK1-importin interaction, Matchmaker™ GAL4 Two-Hybrid System 3 (Clontech, USA) was used. For ZoCDPK1-library interaction Matchmaker™ Gold Yeast Two-Hybrid System (Clontech, USA) was used.

### Preparation of cDNA from RNA using SMART™technology

Total RNA was isolated from leaves of salinity stressed ginger plants by TRIzol® method (Invitrogen, USA). From this Poly (A) RNA was isolated from total RNA with NucleoTrap® mRNA kit (Clontech, USA) as per the manufacturer's protocol. 1 μg of poly (A) RNA was reverse transcribed using SMART M-MLV reverse transcriptase and further converted to ds cDNA using SMART technology as per manufacturer's protocol (Clontech, USA). The ds cDNA was purified using CHROMA SPIN TE-400 column to select DNA molecules greater than 200 bp.

### Construction of *Z. officinale* yeast two-hybrid library

For ginger library construction Y187 yeast competent cells were prepared according to the protocol of the Yeastmaker Yeast Transformation System 2 (Clontech, USA). About 20 μL of the ds cDNA of ginger (2–5 μg) and 3 μg of pGADT7 were co-transformed into the yeast strain Y187 to construct the ginger Y2H library according to the protocol mentioned in Make Your Own “Mate & Plate™” Library System (Clontech, USA). To determine the complexity of the library, 100 μL of 1/10 and 1/100 dilutions of transformed cells were spread on SD/−Leu (synthetically defined medium lacking leucine) 100 mm agar plates. After incubation at 30°C for 3–4 days, the number of colonies on dilution plates was counted and the transformation efficiency was calculated. To identify the titer of the constructed ginger Y2H library, 10 μL of library aliquot was taken out and diluted to 1/100, 1/1000, 1/10000, and 1/1000000. The last two dilutions were spread in duplicate on SD/−Leu 100 mm agar plates, and the library titer was calculated according to the colonies appearing (Fu et al., 2013).

### The bait and prey constructs

In a Matchmaker GAL4-based two-hybrid assay, a bait protein is expressed as a fusion with the Gal4 DNA-binding domain (DNA-BD), while libraries of prey proteins are expressed as fusions with the Gal4 activation domain (AD). To this end four different bait vectors namely CD1-F (Full length ZoCDPK1), CD1-VK (NV-K domain of ZoCDPK1 only), CD1-JRV (JD-CaMLD-CV of ZoCDPK1), and CD1-RV (CaMLD-CV of ZoCDPK1) were made by amplifying the appropriate portion of ZoCDPK1 by PCR. The PCR was carried out in a 50 μL reaction mixture containing 100 ng of plasmid DNA (containing ZoCDPK1 clone), 5x Phusion High-Fidelity Reaction Buffer, 200 μM of each dNTPs, 1.25 U of Phusion DNA polymerase (NEB, England) and 20 pmol each of forward and reverse primers (Vivek et al., 2013). The primers used were CD1YF1 (5′-CATGGAGGCCGAATTCATGGGAAATTCCTTCGTCTGCTGC-3′) and CD1YR1 (5′- GCAGGTCGACGGATCCTGACGGATGGTTTGCGTCTTTATC-3′) for CD1-F; CD1YF1 and CD1YR3 (5′-GCAGGTCGACGGATCCCAACCATGGATGTTCCAACACCTG-3′) for CD1-VK; CD1YF2 (5′-CATGGAGGCCGAATTCCAAAATGCCAAGAAGGCTTCCAAT-3′) and CD1YR1 for CD1-JRV; CD1YF3 (5′-CATGGAGGCCGAATTCGTTATCAGAGACATGTTTAGGTTA-3′) and CD1YR1 for CD1-RV. They were then cloned in-frame into the *Eco*RI and *Bam*HI sites of pGBKT7 (Clontech) and expressed as a fusion with the yeast GAL4 DNA-BD.

Two different prey constructs were used in *in vitro* interaction studies. For one-to-one interaction, coding region of ZoIMPα was amplified by PCR in a 50 μL reaction mixture containing 100 ng of plasmid DNA (containing ZoIMPα clone), 5x Phusion High-Fidelity Reaction Buffer, 200 μM of each dNTPs, 1.25 U of Phusion DNA polymerase (NEB, England) and 20 pmol of IMPYF (5′- GAGCGCCGCCATGGAGATGTCGCTCCGGCCGAGCGAGAGG-3′) and IMPYR (5′- GCAGGTCGACGGATCCCTTATCGCCGATCCGGCTGCGGAG-3′) and cloned in-frame into the *Nco*I and *Bam*HI sites of the activation domain vector pGADT7 (Clontech). All constructs were verified by direct DNA sequence analysis using T7 primer, DNA-BD primer and AD primer. For library screening, ginger cDNA library (salinity stress induced) cloned in pGADT7 and transformed in yeast strain Y187 were used as prey.

### ZoCDPK1-importin-α interaction

To check the interactions between different domains of ZoCDPK1 and ZoIMPα protein, AD and BD vectors containing the desired plasmids were co-transformed into yeast strain AH109 harboring two reporter genes (HIS3 and β-galactosidase) by lithium acetate method. Separate co-transformations were carried out for all the four bait constructs. In each co-transformation, 5 μg of both AD (CD1-F/CD1-VK/CD1-JRV/CD1-RV) and BD plasmids and 100 μg of denatured carrier DNA (ssDNA) were combined in a pre-chilled sterile 1.5 mL microcentrifuge tube and 100 μL of yeast competent cells were added to it and mixed gently. To each reaction mix 600 μL of TE-LiAc/PEG solution was added and mixed by vortexing. All four reaction mixtures were incubated at 30°C for 30 min with gently mixing on every 10 min by tapping. After incubation 70 μL DMSO was added to each tube and was placed in a 42°C water bath for 15 min. The tubes were centrifuged at 3000 rpm for 15 s. The supernatant was removed from each tube and the pellets were re-suspended in 0.5 mL 1x TE buffer separately. The centrifugation and re-suspension steps were repeated two more times and the final pellet in each tube was re-suspended in 200 μL of 1x TE buffer separately. Yeast cells carrying both the plasmids were selected on synthetic medium lacking Leu and Trp (SD-Leu–Trp-). The plates of all the four co-transformation reactions were incubated upside down at 30°C until colonies appear (3–5 days).

AH109 contains integrated copies of ADE2, HIS3, and lacZ (MAL1) reporter genes under the control of distinct GAL4 upstream activating sequences (Kamei et al., 2001) and TATA boxes. These promoters yield strong and very specific responses to GAL4. For all the four reactions, the positives colonies from the double drop out plates were then streaked onto an SD medium lacking Leu, Trp, and His (Leu-Trp-His-) and with 15 mM 3-AT to determine the expression of HIS3 nutritional reporter.

The β-galactosidase expression of the His^+^ colonies was analyzed by filter-lift assays as described by the manufacturer (Clontech, USA). In order to do filter-lift assay, single colony from each plate (CD1-F, CD1-VK, CD1-JRV, and CD1-RV) was patched separately on a YPD plate and incubated for 1 day at 30°C. A sterile Whatman No. 3 filter paper was pre-soaked by placing it in 5 mL of Z buffer/X-gal solution in a clean 150 mm plate. Using forceps, a clean, dry filter paper was placed over the surface of the colonies grown on the YPD plate and gently rubbed the filter with the side of the forceps to help colonies cling to the filter. When the filter has been evenly wetted, carefully lifted it off the plate with forceps and transferred it (colonies facing up) to a pool of liquid nitrogen. Using the forceps, the filters were completely submerged. After the filter has frozen completely (~10 s), removed it from the liquid nitrogen and allowed it to thaw at RT. Then carefully placed the filter, colony side up, on the presoaked filter paper by avoiding air bubbles trapped under or between the filters and incubated the filters at 30°C and checked periodically for the appearance of blue colonies. The β-galactosidase producing colonies were identified by aligning the filter to the agar plate using the orienting marks.

### Yeast two-hybrid screening of libraries

To screen proteins from ginger (stress induced) cDNA library interacting with ZoCDPK1, the bait protein with complete coding sequences of ZoCDPK1 cloned into the *Eco*RI and *Bam*HI sites of pGBKT7 (Clontech, USA) and expressed as a fusion with the yeast GAL4 DNA-BD (Fu et al., 2013). For this purpose, the complete ORF of ZoCDPK1 was amplified from cDNA by PCR using the primers, CD1YF1 and CD1YR1. Before screening, the bait protein was tested against the autoactivity and toxicity of ZoCDPK1 in the absence of a prey ginger library. A concentrated Y2HGold (pBD-ZoCDPK1) culture was mixed with 1 mL of Y187 (pAD-ginger) Y2H library for mating in accordance with the Matchmaker Gold Yeast Two-Hybrid protocol (Clontech, USA). Thirty 150 mm DDO/A (double dropout medium lacking tryptophan and leucine supplemented with Aureobasidin A) plates were used to screen the clones after mating for 3–5 days. All blue colonies were then patched out and allowed to grow on QDO/A (quadruple dropout medium lacking adenine, histidine, tryptophan and leucine and supplemented with Aureobasidin A) plates (Fu et al., 2013). The blue colonies were screened twice on QDO/X/A plates to rescue additional library plasmids and eliminate false positives. Meanwhile, the bait plasmid (pGBKT7-ZoCDPK1) was co-transformed into Y2HGold with the prey plasmid (pGADT7-T) to serve as negative control, respectively (Fu et al., 2013; Gao et al., 2016). The AD/library was further screened by yeast colony PCR using Matchmaker Insert Check PCR mix (Clontech, USA) for eliminating duplicates. For PCR of each colony, a very small amount of cells were picked using a toothpick and placed on a tube containing 25 μL of PCR grade water. To this 25 μL of Matchmaker Insert Check PCR Mix was added and mixed up and down for 5 times. The thermal cycling parameters were as follows: Initial denaturation at 94°C for 1 min followed by 30 cycles of 98°C for 10 s; 68°C for 3 min. The PCR products were analyzed by electrophoresis on a 1% agarose/EtBr gel. The products were further cloned and sequenced. The sequences were analyzed using the NCBI BLASTN and BLASTP programs.

## Results

### Model of unimolecular structure of CDPK from *Zingiber officinale*

**Figure 1:**
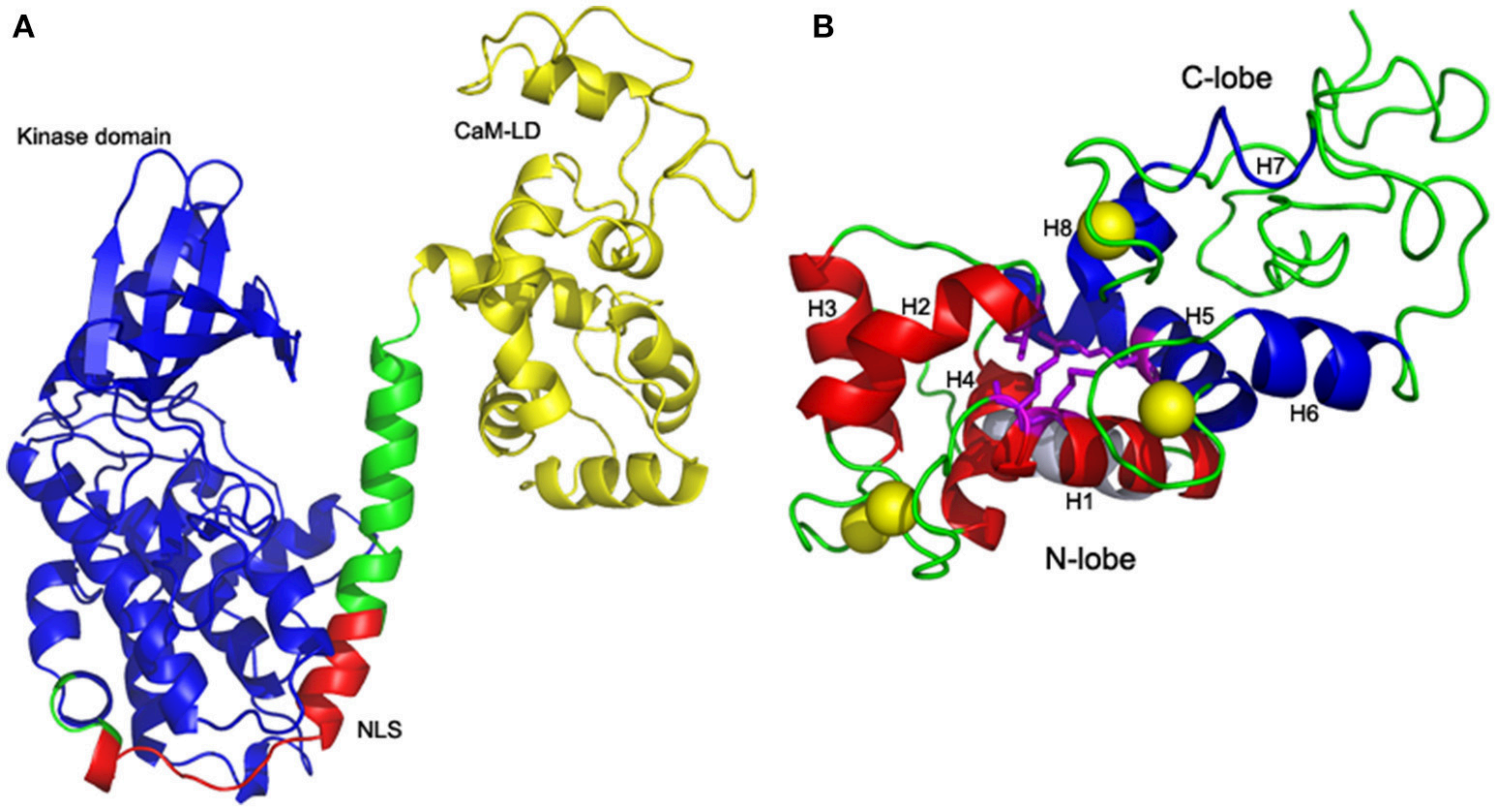
**Homology model of ZoCDPK1. (A)** Mean structure obtained after restrained energy minimization (variable regions are not shown). The color coding is as follows: Kinase, blue; CaM-LD, yellow; JD, green; NLS, red. The terminal catalytic (blue) domain and CaM-LD (yellow) domains are linked by a JD whose NLS region (red) is exposed. The active site of the kinase is spatially away from the NLS. **(B)** Four-helix bundle EF-hand domains: Interdomain communication between N (red) and C (blue) lobes within the CaM-LD polypeptide in the presence of Ca^2+^ (yellow). The 8 helices (H1-H8) of CaM-LD are labeled. The lobes interact predominantly via residues in helices h1 (N-lobe) and h5 (C-lobe) with contributions from residues in h2 (N-lobe) and h8 (C-lobe).

ZoCDPK1 with a typical core domain arrangement of a Ca^2+^ regulated kinase, also possesses a bipartite NLS in its JD. As structural details of full length CDPKs were very much limited, two templates 2BDW and 2AAO were selected for homology modeling as they showed significant homologies with kinase and CaM-LD of ZoCDPK1 respectively. The alignment of sequences of both the templates with ZoCDPK1 is shown in Figure S1. Among the five models generated by MODELLER for ZoCDPK1, the minimum energy structure was selected for further analysis. PROSA2003 quality check shows that the model of the ZoCDPK1 generated is of good quality (Figure 1A), where interaction energy of each residue with the residual of the protein is negative (*Z*-score: –2.89). The proposed structure has core domains with the terminal catalytic (blue) and CaM-LD (yellow) domains linked by a JD, which has mainly an irregular structure (Zheng et al., 2010). The structure of the regulatory domain shows the expected four-helix bundle EF-hand domains, “N” and “C” lobes (Figure 1B). The JD can be divided into three segments. The first is the bipartite NLS bearing region, which may be responsible for its nuclear localization by binding with importin. The second is a pseudosubstrate autoinhibitory region which blocks the substrate binding site of the kinase domain in the inactivated state. Finally there is a CaM-LD binding domain, which involves in the activation of the protein in presence of Ca^2+^.

**Figure 2:**
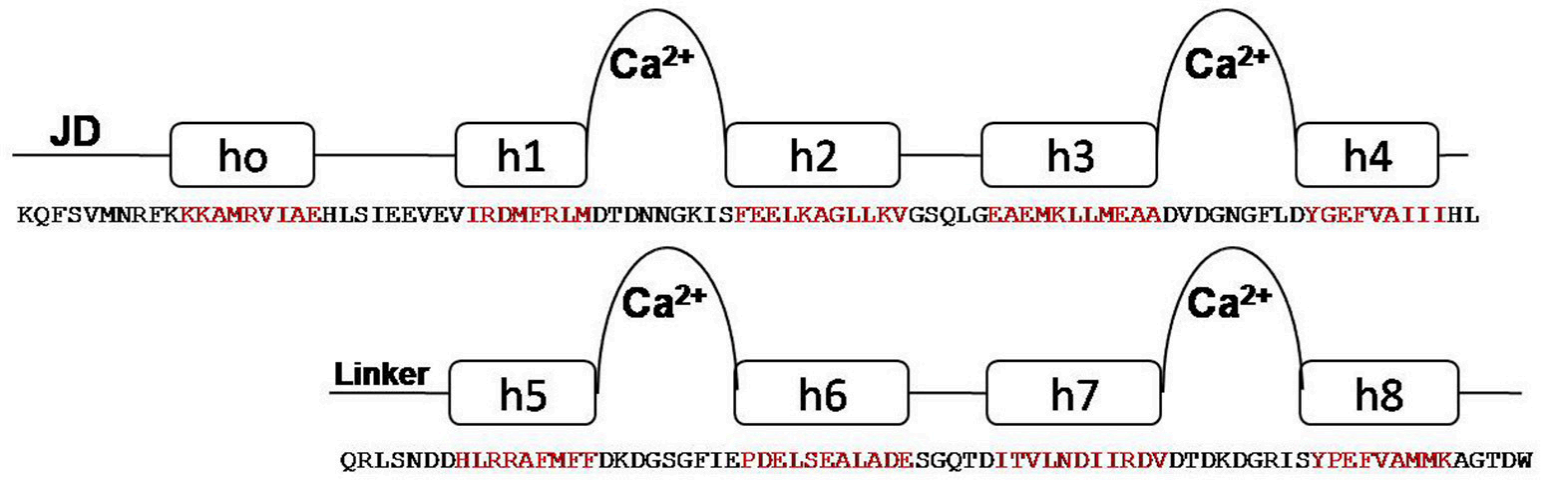
**Structural organization of CDPK regulatory apparatus–amino acid sequences and secondary structure**. The structure can be divided into three segments: The h4-helix of the second EF hand and the h5-helix of the third EF hand form the bookends, with a linker in the middle.

One striking feature of the structure is the interaction between the two lobes of the CaM-LD. The interface between the C-lobe and the N-lobe is shown in detail in Figure 1B. The lobes interact predominantly via residues in helices h1 (N-lobe) and h5 (C-lobe) with additional contributions from residues in h2 (N-lobe) and h8 (C-lobe). It involves both hydrophobic interactions (e.g., Ile369 and Met372 in h1 with the aromatic ring of Phe445 in h5), and electrostatic interactions (e.g., E364 of h1 having H-bonding with Arg443 of h5). Also, Val396 in h2 contributes significant contacts to the aromatic rings of both Arg443 and Tyr494 (in h5 and h8, respectively). The structural organization of the regulatory apparatus of ZoCDPK1 is shown in Figure 2. The helix joining N- and C-lobe emerges from the second EF hand to run almost antiparallel to the JD helix but is comparatively shorter by the length of the autoinhibitory segment. It can also be divided into three segments: The h4-helix of the second EF hand and the h5-helix of the third EF hand form the bookends, with a linker in the middle.

### Three-dimensional model of interaction of *Zingiber* CDPK with importin-α

**Figure 3:**
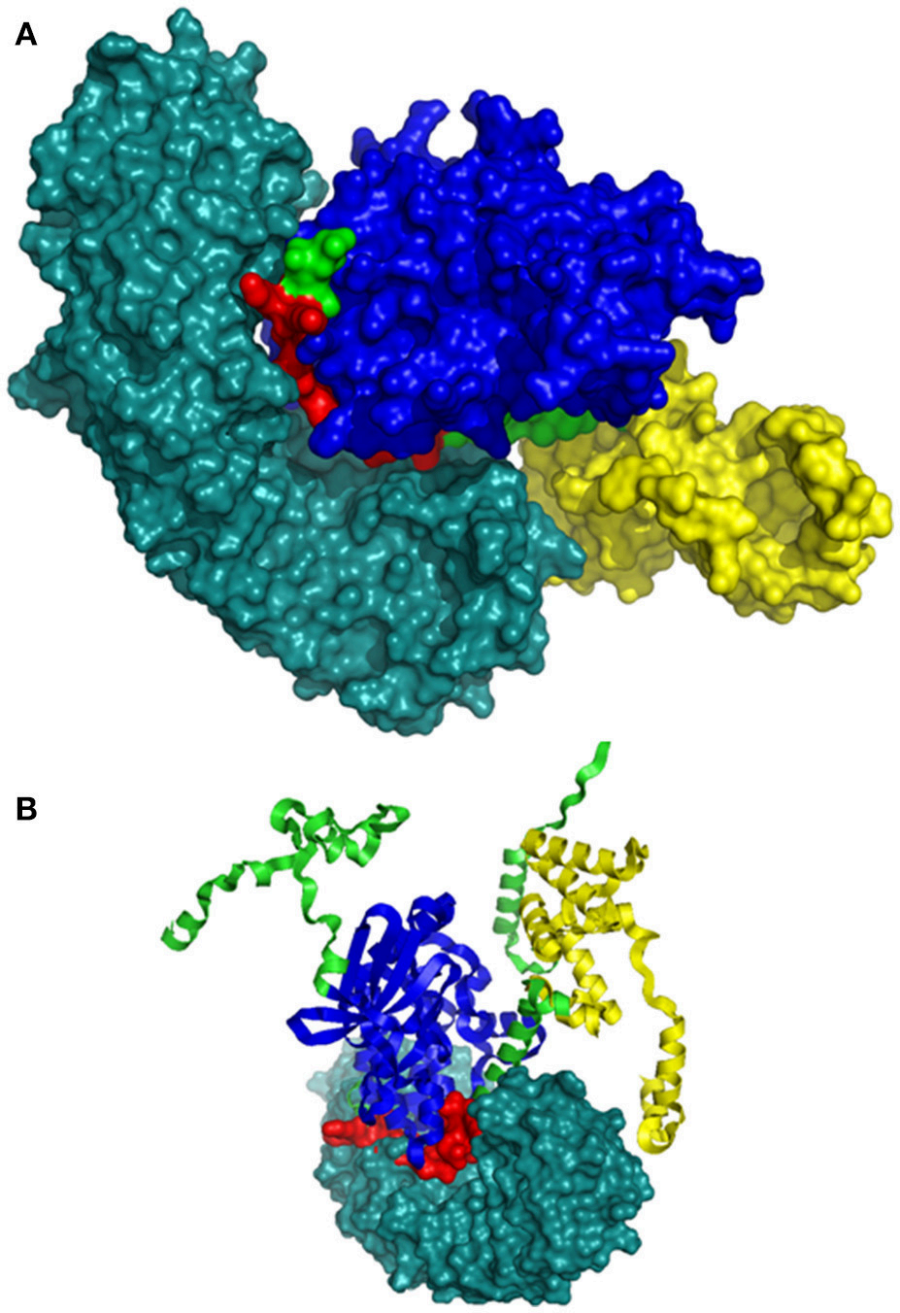
**Model of interaction between ZoCDPK1 and importin α. ZoCDPK1/Importin complex model, showing the interaction partners and their domains**. Importin binding is not interfering with the kinase and the regulatory domain activity. Importin is shown in deep teal and the rest color coding is same as that in Figure 1. **(A)** and **(B)** are respectively the lateral and top view of ZoCDPK1-importin α interaction. In **(B)**, ZoCDPK1 is represented using ribbon model.

**Figure 4:**
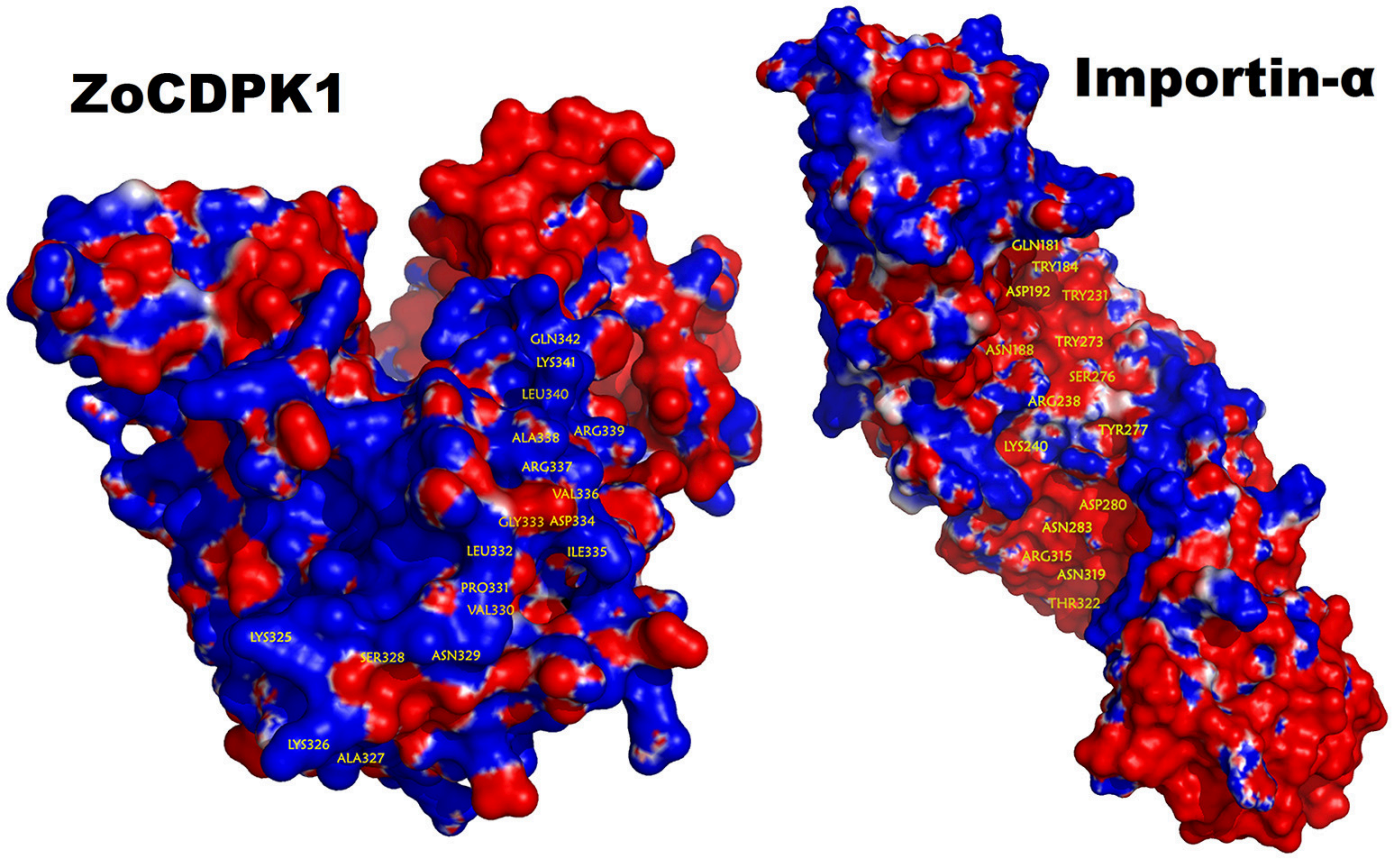
**Electrostatic potential mapped on the molecular surface of the ZoCDPK and of the importin-α protein**. Red and blue represent negative and positive potential calculated using APBS. Charged amino acids on the protein surface.

The bipartite NLS motifs are known to bind importins, the shuttle proteins of the nuclear transporting machinery, through their positive residues (Imamoto et al., 1995). In order to visualize the interaction between ZoCDPK1 and importin, a binary complex was built by protein docking method (Figure 3), using the structure of importin obtained from importin-NLS peptide complex (PDB ID: 1PJN). The NLS region of the JD is exposed in the ZoCDPK1 three dimensional models (Figure 1A; red region) and appears amenable to interact with its cognate receptors or importin. The active site of the kinase is spatially away from the NLS, and consequently the binding of importin at the NLS may not block the catalytic function of the activated kinase. From the structural features of importin-ZoCDPK1 (Figure 3B) also, it was found that importin binding is not interfering with the kinase and the regulatory domain activity. The electrostatic surface potentials of the two proteins were calculated by solving the Poisson–Boltzmann equation and it was found that the interaction surfaces of the two proteins are clearly complementary (Figure 4). We have compared the residues in importin bound to NLS peptide [PDB: 1PJN] with the residues of importin interacting with the NLS region of *Zingiber* CDPK. The interacting residues of importin are *Gly150, Thr151, Ser152, Trp184, Asn188*, Asp192, *Trp231, Asn235, Arg238*, Asp264, Glu266, *Trp273*, Asp280, *Thr311, Arg315*, Asp325, Asp326, Lys348, Thr349, *Asn350, Lys353, Glu354*, Ala389, Asp390, Phe391, Lys392, Thr393, Gln394, Lys395, *Glu396*, Lys432, Asp433, where the residues in italics are also present in the importin of 1PJN.

### Importin-α isolation from ginger and its analysis

As the attempts to demonstrate importin-α binding with the whole kinase, where the NLS in the JD is sandwiched between the catalytic and the CaM-LD domains, was successful *in silico*, further experiments were conducted to study the *in vivo* one-to-one interaction between them too through Gal4-based two-hybrid system. The cDNA clone of ginger importin-α (ZoIMPα) fragment of approximately 1.6 kb was amplified by RT-PCR using IMPF and IMPR primers designed based on EST sequences as described in the Materials and Methods. Sequence analysis of ZoIMPα cDNA shows that it is a full-length cDNA, which is 1593 bp in size and encodes a protein consisting of 531 amino acid residues with a predicted molecular mass of about 58.67 kDa and isoelectric point of 5.09. ZoIMPα contains an N-terminal 58 amino acid IBB domain (a highly basic domain responsible for importin-β binding) and an NLS-binding domain built of four armadillo (ARM) repeats respectively at 115–158, 159–200, 242–284, and 326–369 as revealed by SMART and PROSITE analysis (Figure S2). ZoCDPK1 showed highest homology with importin-α from *Ricinus communis* (83% amino acid identity, GenBank accession number XP_002512485). Other close homolog include importin-α-1 from *Glycine max* (82% identity, XP_003536050) and importin-α-1 from *Vitis vinifera* (82% identity, XP_002282816).

### ZoCDPK1 interacts with ZoIMPα through its junction domain

**Figure 5:**
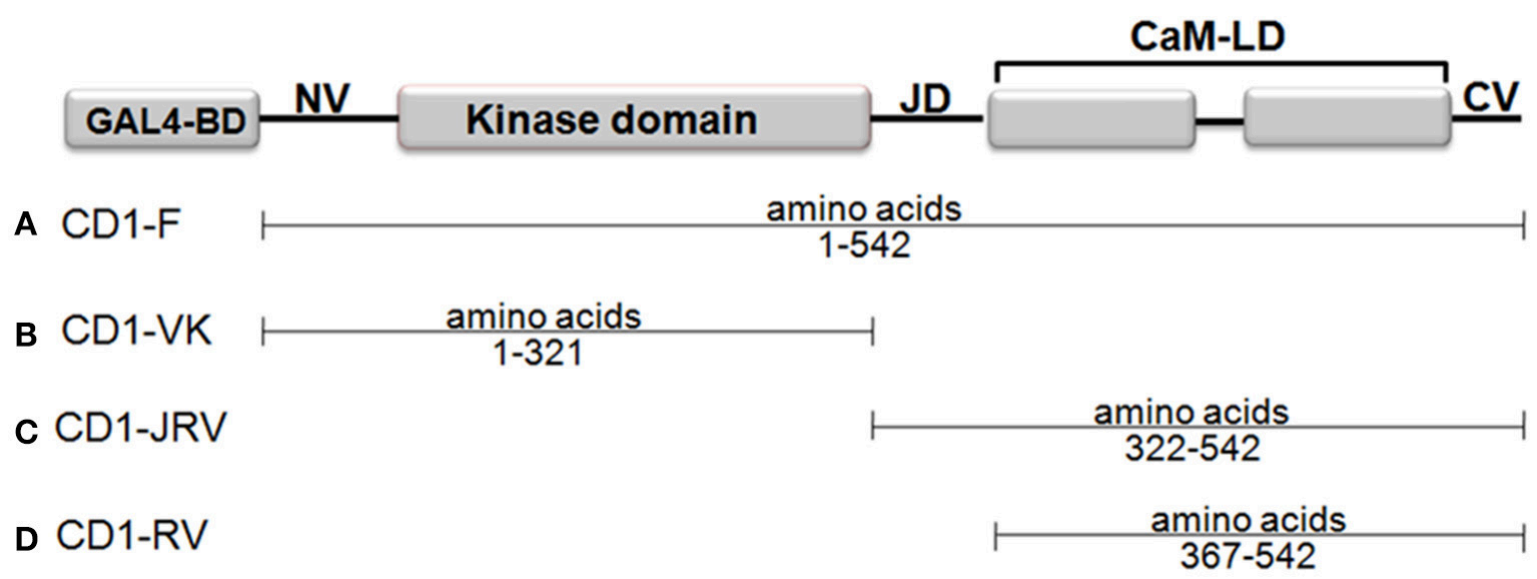
**Domains of ZoCDPK1 that were fused to the Gal4 DNA binding domain and employed as baits in the yeast two-hybrid system**. The N-terminus of each of portion of ZoCDPK1 was fused to the C-terminus of the Gal4 DNA binding domain. The name given for ZoCDPK1 baits is listed to the left of the construct.

**Figure 6:**
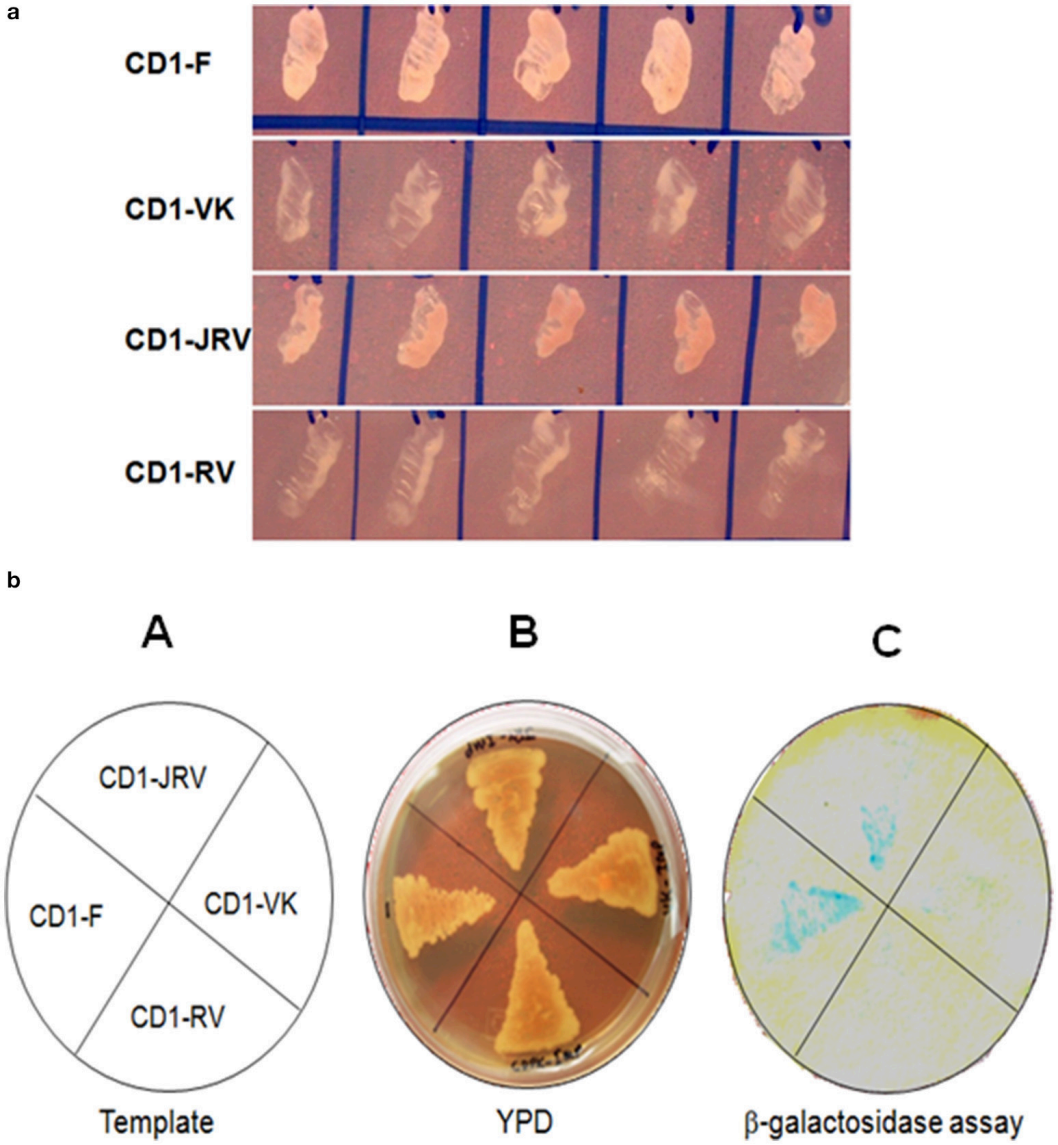
**(a)** Yeast two-hybrid system-based interaction between ZoCDPK1 baits and ZoIMPα on triple DO medium. Yeast cells containing the ginger importinα and the designated bait growing on medium lacking leucine, tryptophan, and histidine. **(b)** ZoCDPK1/ZoIMPα interaction and the domain of ZoCDPK1 necessary for its interaction with ZoIMPα analyzed by yeast twohybrid assay. (A) The template showing the clones streaked (B) Transformants on YPD media. (C) The results from β galactosidase filter lift-assay. The full length CDPK1 protein (CD1-F) as well as JD bearing construct (JRV) produced interaction with ZoIMPα, whereas ZoCDPK1 constructs without JD (CD1-VK and CD1-RV) produced no significant interaction with importin-α.

Four different ZoCDPK1 bait (pGBKT7) constructs were created to dissect the nature of the protein-protein interaction (Figure 5) with importin-α. The use of different portions of ZoCDPK1 gave insight into which part of ZoCDPK1 interacted with the prey protein. All bait constructs were tested for their ability to auto-activate the adenine (ADE2) nutritional reporter. The complete ORF of ZoIMPα gene cloned in a yeast AD vector (pGADT7) was used as prey. AD and BD constructs were co-transformed in yeast AH109 cells and then screened on double (Leu- and Trp-) and triple [(Leu-, Trp- and His-) + 10 mM 3-AT] DO-SD media (Figure S3 and Figure 6a respectively). The interaction was also confirmed through β-galactosidase assay (Figure 6b). As shown in Figure 6b, the full length CDPK1 protein as well as JD bearing construct (JRV) produced interaction with ZoIMPα, whereas ZoCDPK1 constructs without JD (VK and RV) produced no significant interaction with importin-α.

### Screening of interacting partners of *Zingiber officinale* CDPK1 using yeast two hybrid assay

**Figure 7:**
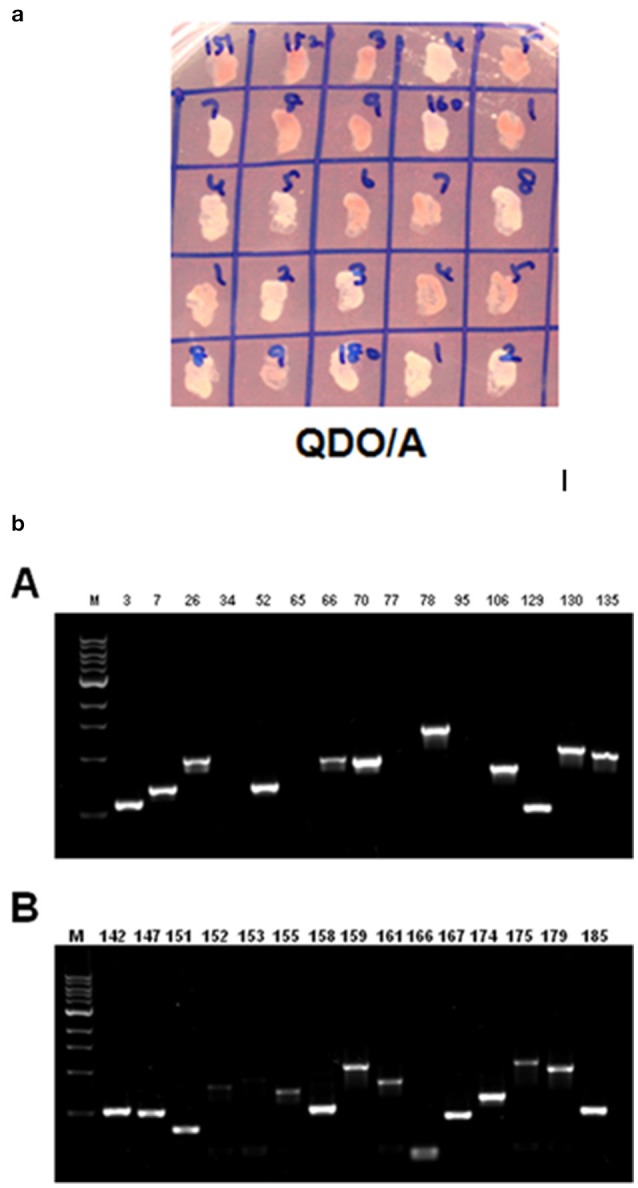
**(a)** Yeast two-hybrid system-based screening of ginger CDPK1 interactors using the ginger cDNA library. Yeast cells on QDO/A plates (quadruple dropout medium i.e., selection medium with all essential amino acids except four amino acids (adenine, histidine, tryptophan, and leucine) and supplemented with Aureobasidin A). **(b)** PCR amplification using AD specific primers on ZoCDPK1-ginger Y2H library interacting colonies on selection medium. The selection was done on quadruple dropout medium lacking adenine, histidine, tryptophan, and leucine and supplemented with Aureobasidin A.

ZoCDPK1 is clearly not an upstream gene of Ca^2+^ signaling pathways and also has strong nuclear translocation. So it was speculated that the function of CDPK could be related to transcriptional regulation. It is known to be associated with a number of proteins in exerting its functional biological roles. In order to screen the interacting partners of ZoCDPK1, Gal4-based yeast two hybrid assay system was used (Clontech, USA). *Z. officinale* cDNA library (salinity stress induced) cloned in pGADT7 vector was used as prey component and ginger CDPK1 cloned in pGBKT7 was used as bait. Approximately eight million transformants were screened and colonies expressing putative ZoCDPK1-interacting proteins were selected early on DDO/A (Figure S4) plates and further on QDO/A plates (Figure 7a). The positive colonies were used as templates for PCR analysis using AD specific primers (Figure 7b). The amplified cDNA was subsequently sequenced, and the identity of the prey proteins was determined by analyzing the cDNA sequences using BLAST and PSI-BLASTX searches.

### Identification of putative positive protein-protein interactions

The prey cDNA from all 25 putative positive yeast clones (including redundant amplicons) from the yeast two-hybrid experiment was successfully amplified using PCR. Of these 25 clones, 22 samples of amplified cDNA yielded adequate sequencing results and credible BLAST analysis data. The sequencing data were further analyzed and sorted into groups based on cDNA insert size to reduce the number of duplicate clones. BLAST analysis from sequence data identified the following proteins: NAC transcription factor, GATA transcription factor, EF1α, Malate dehydrogenase, 40S ribosomal protein, Gibberellin 20 oxidase, Ubiquitin conjugated enzyme (E2), GRF1-interacting factor, Phospholipid hydroperoxide glutathione peroxidase, and somatic embryogenesis receptor like kinase (Table 1).

**Table 1:** **Putative positive proteins that interact with ZoCDPK1 identified by using the yeast two-hybrid system**.

**Clone**	**Gene**	**Function**
3, 129	Gibberellin 20 oxidase	Transcription
7, 52, 174	40S ribosomal protein	Component of ribosome (Initiation of translation)
26, 66, 70, 135	NAC transcription factor	Stress response
79	Malate dehydrogenase	Metabolic pathways (TCA cycle, gluconeogenesis etc)
106	Ubiquitin conjugated enzyme	Protein degradation via proteasome
130, 151, 179	GATA transcription factor	Gibberellin signaling
142, 147, 158, 67, 185	EF-1α	Eukaryotic translation; Nuclear export of proteins
161	GRF1-interacting factor	Cell expansion in leaf
174	Phospholipid hydoroperoxide glutathione peroxidase	Enzyme involved in glutathione metabolism
175	Somatic embryogenesis receptor like kinase	Plant development; hormone signaling; defense response

*The function of each gene was determined using information obtained from BLAST and PSI-BLASTX searches (National Center for Biotechnology Information:* http://www.ncbi.nlm.nih.gov/blast/Blast.cgi*)*.

## Discussion

### ZoCDPK1 full length model justifies current activation mechanism of CDPK

In order to get insight into the mechanism of activation of ginger CDPK, a full length molecular model of ZoCDPK1 was generated. The templates chosen for full length modeling of ZoCDPK1 showed optimum homology and the PROSA2003 *Z*-scores of the model and template structures were more or less similar which indicated the quality of the model. The regulatory domain of ZoCDPK1 full length model was found to mimic closely with the crystal and modeled structure of CaM-LD of CDPK1 (CPK-1) from *Arabidopsis* (Chandran et al., 2006). One striking feature of the structure is the interaction between N and C lobe of the CaM-LD and such a type of interaction is essential for the activation of CDPKs. Interestingly, the NMR model of the CaM-LD of soybean CDPKα and AtCDPK1 of *Arabidopsis* also reveals an inter-domain contact, but surprisingly, the interacting residues are not completely similar (Weljie and Vogel, 2004; Chandran et al., 2006). In CDPKs, the Ca^2+^-affinity of the C-lobe is significantly greater than that of the N-lobe. It suggests that there are differential roles of the two lobes in the activation of CDPKs, with the Ca^2+^-loading of the N-lobe as the likely trigger for physiological CPDK activation. In canonical CDPKs, at basal cytosolic Ca^2+^ levels high affinity binding sites in the C-lobe are occupied and it interacts with the junction domain, but the kinase remains in an auto-inhibited state. Activation occurs when Ca^2+^ levels rise to fill the two weaker affinity binding sites in the N-lobe, which alters its interaction with the C-lobe triggering a conformational change that leads to the exposure of the catalytic site and activation (Christodoulou et al., 2004; Vivek et al., 2013). Hence such a type of inter-domain communication is mandatory for the activation of CDPKs. In the present CDPK structure, the interaction between C-terminal segments of the JD region with residues in the C-terminal lobe of the CaM-LD is not significant and moreover, a closer association between N-lobe of CaM-LD and JD was noticed. Considering all the above observations, it may be concluded that the molecular model of ginger CDPK generated in the study represents the structure of an active CDPK.

### Nuclear translocation of ZoCDPK1 is mediated through importin-α binding to its JD

The prediction of importin mediated nuclear localization of ZoCDPK1 was substantiated by molecular docking studies with importin-α, and it was further confirmed by *in vivo* interaction studies. Importin-α is the nuclear import receptor that recognizes cargo proteins carrying conventional basic monopartite and bipartite nuclear localization sequences(NLS) and facilitates their transport to the nucleus. A known mechanism for regulating the activity of NLS is masking it in order to prevent its recognition by importins (Jans, 1995; Vandromme et al., 1996). It is interesting to note that by virtue of having the NLS in the JD, this target group of CDPKs can have their NLS masked by the catalytic domain in the inactive form of the kinase. But at saturating concentrations of Ca^2+^, the NLS may be exposed due to the removal of interaction between JD and kinase. The template chosen for regulatory domain modeling was a Ca^2+^ bound one; hence our model describes the structure of an active CDPK as indicated by the exposed nature of NLS. Moreover, as described earlier, the N-lobe of CaM-LD of ZoCDPK1 shows association with JD, which is a feature of active CDPK. So it may be possible that ZoCDPK1 is interacting with importin-α at its active state and further translocated to the nucleus which in turn justifies its involvement in multiple stress signaling.

Electrostatic and hydrophobic interactions appear to be important stabilizing forces determining the local structure of JD, which in turn influences the orientation of NLS and ultimately the binding within the importin protein. Interestingly, it was consistent with the expectations for intermolecular interactions where the hydrophobic forces stabilize the protein–protein interaction and the electrostatic forces orient the molecules and confer specificity to the interaction. The ZoCDPK1 homology model illustrates that Lys325, Lys326, Arg337, Arg339, and Lys341 are located in the N and C terminal of NLS. Within this region, the columbic interactions among Asp192, Asp264, Glu266, Asp280, Asp325, Asp326, Glu354, Asp390, Glu396, Asp433 of importin and the orientation of hydrophobic residues may be vital for appropriate ZoCDPK1-importin interaction and nuclear localization signaling.

The result of two-hybrid analysis revealed that the amino acid sequences present in the JD-containing fragment of ZoCDPK1 are responsible for its interaction with importin-α. The 18-residue bipartite NLS present in the first part of JD is responsible for binding with importin-α. This observation well-correlates with the *in silico* interaction data in which importin clearly interacts with the NLS of ZoCDPK1. Even though the presence of an NLS has been observed in many CDPKs, its functional nature in CDPKs was not studied so far. The present study is the first report regarding the availability of a biologically functional NLS in CDPKs.

(Raichaudhuri et al., 2006) reported that in CDPKs, the NLS in the JD is always coupled with non-consensus Ca^2+^ binding EF-hand domains in their CaM-LD. Due to this atypical Ca^2+^ binding property, such CDPKs invariably requires an activation trigger like “binding with importin” to their NLS region. Hence they are supposed to be functional only in the nucleus. The kinase activity of these CDPKs on any of the available substrates is not reported so far too (Wu et al., 1996). ZoCDPK1 (and many other recently reported CDPKs) also has an NLS sequence in the JD and consensus sequence in the EF-hand domains of CaM-LD. In ZoCDPK1, the NLS is biologically functional too. Moreover, the Importin-ZoCDPK1 complex structure (*in silico* model) shows that importin binding does not interfere with its kinase activity as the kinase and regulatory domains are spatially away from importin. Hence, for ZoCDPK1, the binding of importin is not required for the activation of protein. ZoCDPK1 also showed *in vitro* kinase activity against Histone III-S substrate (unpublished data). Considering all the above factors, it may be possible that ZoCDPK1 also has an activation mechanism similar to that of canonical CDPK.

### ZoCDPK1 interacts with many stress responsive proteins

The yeast two-hybrid system has been used effectively for the identification of *in vivo* substrates for protein kinases. A mutated MEK1 (Raf phosphorylation sites changed to alanine) was able to interact with Raf (both proteins from HeLa cells) in the yeast two-hybrid system, whereas non-mutant MEK1 was not (Wu et al., 1996). The mutated phosphorylation site helped to stabilize the interaction. This is presumably due to the formation of a non-productive complex (Wu et al., 1996; Patel et al., 1999). The success of this approach depends on the fact that physical interactions between a protein kinase and its protein substrate determine a large portion of its specificity (Smith et al., 1999). As CDPKs have been functionally characterized as important regulators involved in plant responses to drought, salt and biotic stresses, identification of targets or substrates become an important task for further understanding the molecular mechanisms of CDPK functions. Increasing evidence for the identification of CDPK substrates supports the notion that CDPKs are multifunctional kinases involved in the regulation of diverse cellular functions (Harper and Harmon, 2005). Efforts have been made to identify CDPK-interacting proteins, such as ABF4 for AtCPK32 (Choi et al., 2005), AtDi19-1 for CPK11 (Rodriguez et al., 2006), StRBOHB for StCDPK5 (Kobayashi et al., 2012), ABF1 and ABF4 for AtCPK4 and AtCPK11 (Zhu et al., 2007), RSG for NtCDPK1 (Ishida et al., 2008), and HSP1 for AtCPK10 (Zou et al., 2010).

ZoCDPK1 is a multiple stress responsive CDPK from ginger and is having predominant nuclear localization during normal and stress conditions. Hence identification of potential interacting partners of this CDPK will lead to a deeper understanding of the functional significance of this Ca^2+^ sensor during stress signal transduction in ginger. In this direction, through yeast hybrid experiment, 10 putative interacting partners (Table 1) were identified from stress induced Y2H library of ginger. The functional roles of these proteins are discussed below.

#### NAC transcription factor

NAC (NAM/ATAF1,2/CUC2) transcription factors are one of the largest families of plant specific transcriptional regulators, and members of the NAC gene family have been suggested to play important roles in the regulation of the transcriptional reprogramming associated with plant stress responses (Nuruzzaman et al., 2013).

#### GATA transcription factor

They are a family of transcription factors characterized by their ability to bind the DNA sequence “GATA” (Ko and Engel, 1993). The GATA-type transcription factors GNC and GNL/CGA1 repress gibberellin signaling downstream from DELLA proteins and PHYTOCHROME-INTERACTING FACTORS (Richter et al., 2010).

#### Elongation factor 1α (EF1α)

EF1α, an essential component of the eukaryotic translational apparatus, is a GTP-binding protein that catalyses the binding of aminoacyl-transfer RNAs to the ribosome (Tatsuka et al., 1992). In addition to its role in translation, eEF1a has been shown to be playing a central role in the nuclear export of proteins (Lee et al., 1999).

#### Malate dehydrogenase (MDH)

MDH is an enzyme that reversibly catalyzes the oxidation of malate to oxaloacetate using the reduction of NAD+ to NADH. This reaction is part of many metabolic pathways, including the citric acid cycle (Minarik et al., 2002).

#### 40S ribosomal protein S18

Ribosomes, the organelles that catalyze protein synthesis, consist of a small 40S subunit and a large 60S subunit. This protein is a component of the 40S subunit and belongs to the S13P family of ribosomal proteins and involved in the initiation of translation.

#### Gibberellin 20 oxidase

Key oxidase enzyme in the biosynthesis of gibberellin that catalyzes the conversion of GA53 to GA20 via a three-step oxidation at C-20 of the GA skeleton (Sasaki et al., 2002). It provides rice cultivars with short, thick culms, raises the harvest index, improves lodging resistance and responsiveness to nitrogen fertilizer, resulting high yields without affecting panicle and grain quality (Spielmeyer et al., 2002).

#### Ubiquitin conjugated enzyme (E2)

They perform the second step in the ubiquitination reaction that targets a protein for degradation via the proteasome (Nandi et al., 2006).

#### GRF1-interacting factor

It is a transcription co-activator that plays a role in the regulation of cell expansion in leaf and cotyledons tissues. The GRFs and their interacting factors (GIFs) acting in the regulation of meristem function, at least partially through the control of cell proliferation. GIFs are involved in the positive regulation of cell proliferation of lateral organs in a functionally redundant manner (Kim and Kende, 2004; Lee et al., 2009).

#### Phospholipid hydroperoxide glutathione peroxidase

This enzyme belongs to the family of oxidoreductases, to be specific those acting on peroxide as acceptor (peroxidases). This enzyme participates in glutathione metabolism (Ursini et al., 1985).

#### Somatic embryogenesis receptor like kinase (SERK)

Receptor-like protein kinases (RLKs) are transmembrane proteins crucial for cell-to-cell and cell-to-environment communications. They are also involved in plant development, somatic embryogenesis, sporogenesis, hormone response, and host defense response (Shi et al., 2012).

All the interactors/substrates identified for ZoCDPK1 is largely involved in stress signal transduction, which re-affirms the multiple stress responsive nature of ZoCDPK1. Modulation of these key proteins needs to be further investigated to understand the underlying mechanism of abiotic stress response mediated via ZoCDPK1.

### ZoCDPK1 may be interacting with NAC transcription factors in the nucleus

Of the different stress related interacting partners identified for ZoCDPK1, NAC TF needs special mention especially in the context of ZoCDPK1 function. NAC proteins are plant-specific TFs which have been shown to function in relation to plant development and also for abiotic and/or biotic stress responses. The cDNA encoding a NAC protein was first reported as RESPONSIVE TO DEHYDRATION 26 (RD26) gene in *Arabidopsis* (Nakashima et al., 2012). Many NAC proteins, including *Arabidopsis* CUC2, have important functions in plant development. Some NAC genes are up-regulated during wounding and bacterial infection (Collinge and Boller, 2001; Hegedus et al., 2003), whereas others mediate viral resistance (Xie et al., 1999). Furthermore, numerous NAC genes are involved in the response of plants to abiotic stresses, such as drought, salinity and submergence (Nakashima et al., 2012).

ZoCDPK1 is rapidly induced by high-salt stress, drought, and jasmonic acid treatment but not by low temperature stress or abscissic acid treatment (Vivek et al., 2013). DRE/CRT/LTRE is a major *cis*-acting regulatory element in ABA-independent gene expression under abiotic stress conditions (Yamaguchi-Shinozaki and Shinozaki, 2005). There are several drought-inducible genes that do not respond to either cold or ABA treatment, suggesting the existence of other ABA-independent pathways in the dehydration stress response (Agarwal et al., 2006; Vivek et al., 2013). The NAC proteins are the prominent candidates in this direction which in cooperation with zinc-finger homeodomain proteins or alone help in transcriptional activation (Agarwal et al., 2006). It was already reported that ZoCDPK1 functions in the positive regulation of signaling pathways that are involved in the response to salinity and drought stress in ginger and is likely operating in a DRE/CRT independent manner (Vivek et al., 2013). The interaction between ZoCDPK1 and NAC TF observed in Y2H experiment further confirms the involvement in DRE/CRT independent pathway. Hence ZoCDPK1 is operating through NAC TF mediated ABA-independent, cold non-responsive pathway in ginger.

## Author contributions

PV performed experiments. KS, MR, and SS assisted the experiments. PV and ES planned the experiments, analyzed the data and wrote the paper. NT assisted in the preparation of manuscript.

### Conflict of interest statement

The authors declare that the research was conducted in the absence of any commercial or financial relationships that could be construed as a potential conflict of interest.
