# Supplementary material for: Calcium-Dependent Protein Kinase in Ginger Binds with Importin-α through Its Junction Domain for Nuclear Localization, and Further Interacts with NAC Transcription Factor
Source: Front Plant Sci. 2017 Jan 13;7:1909. doi: 10.3389/fpls.2016.01909 (PMC5233720; doi:10.3389/fpls.2016.01909)
Supplement: Supplementary file 1 [file DataSheet1.DOCX]

Supplementary Material

**Calcium-Dependent Protein Kinase in Ginger binds with Importin-** **α through its Junction Domain for Nuclear Localization, and further interacts with NAC Transcription Factor**

Vivek P J, Resmi M S, Sweda Sreekumar, Sivakumar K C, Narendra Tuteja and E V Soniya*

**Correspondence:* Soniya E V: [*evsoniya@rgcb.res.in*](mailto:evsoniya@rgcb.res.in)

**Figure S1.**


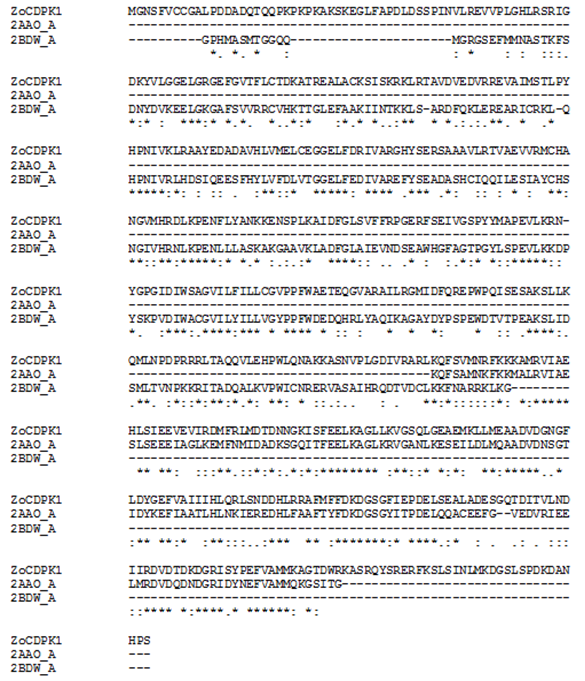


Sequence alignment of ZoCDPK1 with requisite templates used for homology modelling. ClustalW multiple alignment was performed using 2BDW (*C. elegans* auto inhibited kinase domain of CaMKII; Accession 2BDW_A) and 2AA0 (*A. thaliana* CDPK regulatory domain; Accession 2AAO_A) templates.

**Figure S2.**
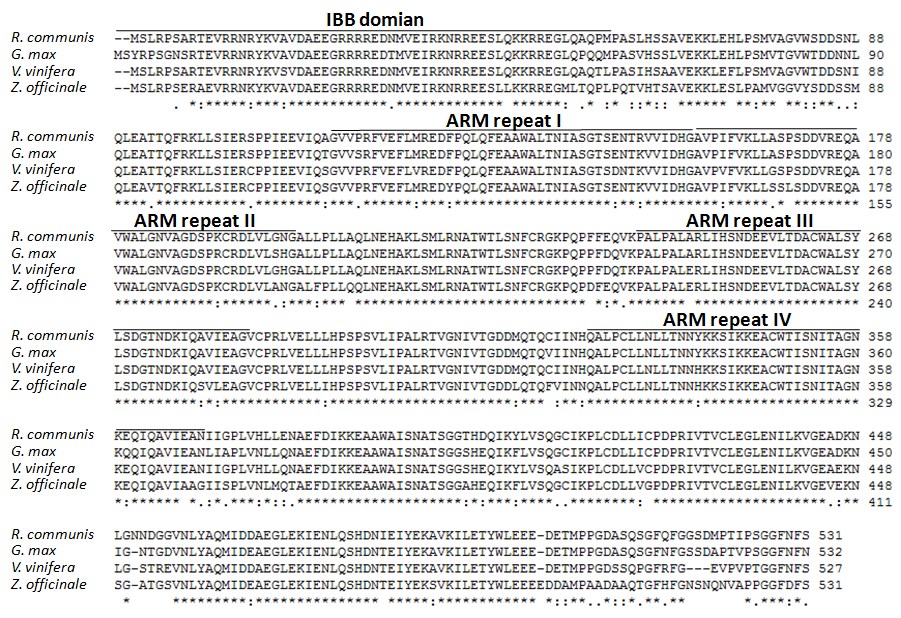


Amino acid sequence alignment ginger IMPα with closely related importins of the database. The amino acid sequence alignment of Zingiber IMPα with that of *R. Communis*, *G. Max* and *V. vinifera* importins (GenBank accession number XP_002512485, XP_003536050 and XP_002282816 respectively). Identical amino acids are indicated by asterisks, and similar amino acids are marked with single dots and colons. IBB domain and four ARM repeats are marked.

**Figure S3**


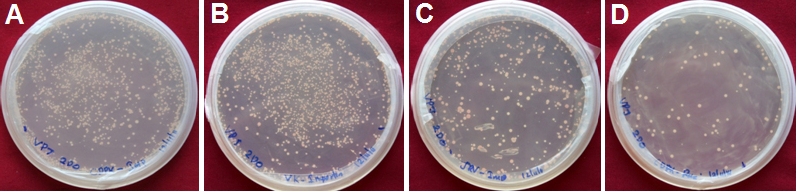

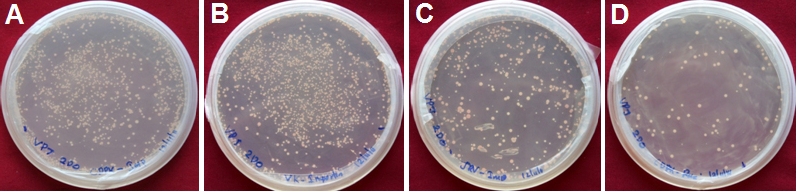


Yeast two-hybrid system-based interaction between ZoCDPK1 baits and ZoIMPα on double DO medium. Yeast cells containing the ginger importinα and the designated bait growing on medium lacking leucine and tryptophan. ZoIMPα interaction with CD1-F (A), CD1-VK (B), CD1-JRV (C) and CD1-RV (D) ginger CDPK bait constructs were shown.

**Figure S4**

**
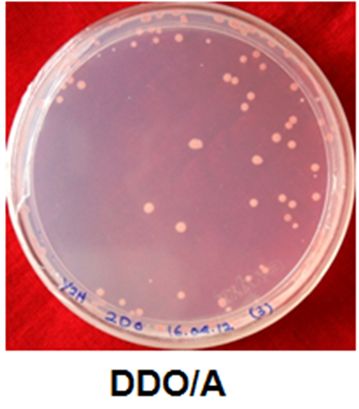
**

Yeast two-hybrid system-based screening of ginger CDPK1 interactors using the ginger cDNA library. Yeast cells containing the ZoCDPK1 and ginger Y2H library on DDO/A plates (double dropout medium lacking tryptophan and leucine supplemented with Aureobasidin A).
